# Effect of L-HSL on biofilm and motility of *Pseudomonas aeruginosa* and its mechanism

**DOI:** 10.1007/s00253-024-13247-7

**Published:** 2024-07-16

**Authors:** Deping Tang, Yanyan Lin, Huihui Yao, Yali Liu, Yanpeng Xi, Mengjiao Li, Aihong Mao

**Affiliations:** 1 School of Biological & Pharmaceutical Engineering, Lanzhou Jiaotong University, Lanzhou, 730070 Gansu China; 2 Gansu Provincial Academic Institute for Medical Research, Lanzhou, 730050 Gansu China

**Keywords:** *P. aeruginosa*, L-HSL, Quorum sensing, Biofilm

## Abstract

**Abstract:**

*Pseudomonas aeruginosa* (*P. aeruginosa*) biofilm formation is a crucial cause of enhanced antibiotic resistance. Quorum sensing (QS) is involved in regulating biofilm formation; QS inhibitors block the QS signaling pathway as a new strategy to address bacterial resistance. This study investigated the potential and mechanism of L-HSL (N-(3-cyclic butyrolactone)-4-trifluorophenylacetamide) as a QS inhibitor for *P. aeruginosa*. The results showed that L-HSL effectively inhibited the biofilm formation and dispersed the pre-formed biofilm of *P. aeruginosa*. The production of extracellular polysaccharides and the motility ability of *P. aeruginosa* were suppressed by L-HSL. *C. elegans* infection experiment showed that L-HSL was non-toxic and provided protection to *C. elegans* against *P. aeruginosa* infection. Transcriptomic analysis revealed that L-HSL downregulated genes related to QS pathways and biofilm formation. L-HSL exhibits a promising potential as a therapeutic drug for *P. aeruginosa* infection.

**Key points:**

*• Chemical synthesis of N-(3-cyclic butyrolactone)-4-trifluorophenylacetamide, named L-HSL.*

*• L-HSL does not generate survival pressure on the growth of P. aeruginosa and can inhibit the QS system.*

*• KEGG enrichment analysis found that after L-HSL treatment, QS-related genes were downregulated.*

**Supplementary Information:**

The online version contains supplementary material available at 10.1007/s00253-024-13247-7.

## Introduction

*Pseudomonas aeruginosa* (*P. aeruginosa*) is a wide range of Gram-negative rod-shaped bacteria in nature and is an important “emerging opportunistic pathogen” worldwide (Gellatly and Hancock 2013). In immunocompromised patients, it can cause various acute and chronic infections, including eye infection (Hilliam et al. 2020), burning infection (Church et al. 2006), pneumonia (Maurice et al. 2018), and bloodstream infection (Moradali et al. 2017) in severe cases. Infections caused by *P. aeruginosa* show a high fatality rate, ranging from 20 to 60% (Tuon et al. 2022). *P. aeruginosa* uses the quorum sensing (QS) system to regulate the expression of many virulence factors, including host damage enzymes (elastase), toxic secondary metabolites (pyomonin, rhamnolipid), and motility (Lee and Zhang 2015). Furthermore, QS promotes *P. aeruginosa* biofilm formation during infection, where bacteria shift from a planktonic state to an adherent growth mode (Aldawsari et al. 2021). Research has been found that biofilm formation and secretion of virulence factors are the major causes of *P. aeruginosa’s* pathogenicity (Ahmed et al. 2019), and biofilm formation significantly increases its antibiotic resistance (Maurice et al. 2018). It is estimated that 80% of chronic infections caused by microorganisms are related to biofilm formation (Jamal et al. 2018). Therefore, a targeted QS system has become a very powerful approach to control its pathogenicity.

QS is a density-dependent communication mechanism between bacterial cells in a population, achieved through the secretion and sensing of small molecules called autoinducers (AI) (Papenfort and Bassler 2016). *P. aeruginosa* consists of three major interconnected systems (LasI/LasR, RhlI/RhlR, and PQS) (García-Reyes et al. 2020). The LasI/LasR and RhlI/RhlR systems are mediated by acyl homoserine lactones (AHLs) signaling molecule, specifically N-(3-oxododecanoyl)-L-homoserine lactone (OdDHL) and N-butyryl-L-homoserine lactone (BHL), respectively (Chadha et al. 2022). The PQS system is a non-AHL-mediated quinolone-dependent system regulated by LasR and RhlR (Lee and Zhang 2015), with the signal molecule being 2-heptane-3-hydroxy-4-quinolone (PQS). Each signal molecule acts as an autoinducer for specific sensing and response systems (Ismail et al. 2016); once these signals reach a “quorum level” (Hmelo 2017), bacteria respond to this change and regulate the expression of QS-related genes and the biofilm formation through cascades of signal transduction within the cell (Chen et al. 2019).

QS inhibitors can minimize antibiotic resistance in conventional antibiotic therapy and are a robust and efficient virulence treatment strategy (Mok et al. 2020). There are two main approaches for obtaining QS inhibitors: natural sources or chemical synthesis. QS inhibitors can target and disrupt the QS system, avoid the pathogenesis of bacteria and any threat to survival, inhibit the expression of virulence factors, and ultimately alleviate infections caused by pathogens (Kalia 2013). In recent years, this field has garnered extensive research attention. Based on this, this study synthesized an AHL analog and studied its effects on *P. aeruginosa* biofilm, extracellular polysaccharide, surface chemical groups of extracellular polymers, and mobility. Additionally, the study explored the mechanism of action through transcriptomic sequencing, hoping to provide suspensions for antibiotic resistance issues in *P. aeruginosa*.

## Materials and methods

### Bacterial strains, culture conditions, and reagents

*P. aeruginosa* PAO1 and *Escherichia coli OP50* were stored in Luria–Bertani (LB) broth medium containing 30% glycerol at − 80 °C and were cultured at 37 °C and 150 rpm in LB broth medium.

*C. elegans* were stored in an S-buffer solution containing 30% glycerol and preserved at − 80 °C; before use, they were quickly and completely thawed and centrifuged at 4000 rpm for 1 min; the supernatant was discarded, and the remaining *C. elegans* suspension was added to a nematode growth medium (NGM) plate, which was coated with *E. coli OP50* using a pipette. The plate was rotated to distribute it evenly, cultured at 20 °C, and passaged three times.

### L-HSL synthesis

**Fig. 1:**
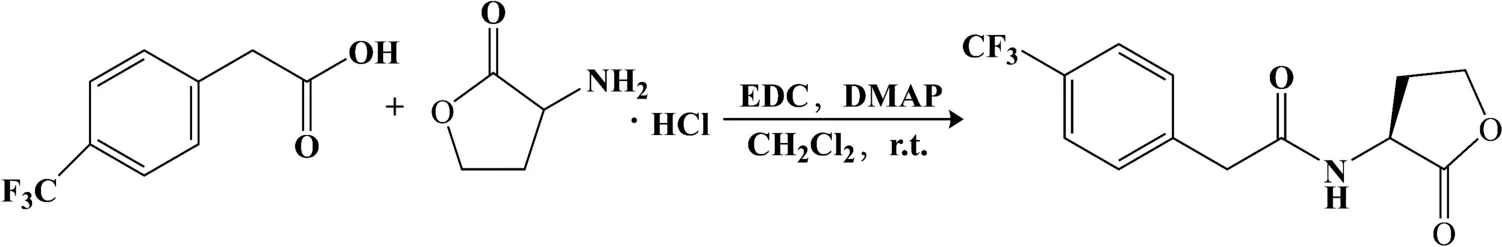
Chemical synthesis process of L-HSL. (S)-(-)-α-amino group-γ-butyrolactone hydrochloride and 4-(trifluoromethyl) phenylacetic acid were reacted at 0 °C for 20 h. L-HSL (N-(3-cyclic butyrolactone)-4-trifluorophenylacetamide) was isolated using the extraction method

For the synthesis of L-HSL based on the structures of the natural signaling molecules OdDHL and BHL, we retained the natural homoserine lactone ring and substituted the acyl side chain to synthesize an AHL analog, named L-HSL. The synthesis method is depicted in Fig. 1. (S)-(-)-α-amino group-γ-butyrolactone hydrochloride provides lactone ring structure, and 4- (trifluoromethyl) phenylacetic acid provides acyl side chain structure. The resulting product was characterized by ^1^H NMR. L-HSL was dissolved in dimethyl sulfoxide (DMSO). The final concentration of DMSO in the suspension was 0.3% (v/v).

### Determination of the growth curves

*P. aeruginosa* was cultured overnight in LB broth medium to logarithmic phase, with a bacterial concentration of 2.8 × 10^9^ CFU/mL, fresh PPGAS medium was then used to dilute the culture to a concentration of 1 × 10^7^ CFU/mL, and the diluted cultures containing L-HSL (final concentration: 0, 10, 100, 200 µM) were transferred to sterile triangular bottles and were incubated at 37 °C and 150 rpm for 24 h. The optical density at OD_600_ nm was measured every 2 h for a total of three replicates (Han et al. 2022).

### Effect of L-HSL on biofilm

#### Effects of L-HSL on biofilm formation

The log phase (2.8 × 10^9^ CFU/mL) bacteria suspension was diluted with fresh PPGAS medium to a concentration of 1 × 10^7^ CFU/mL and were grouped and labeled; 150 µL bacterial suspension containing 0, 10, 100, and 200 µM L-HSL, respectively, was added to a 96-well plate. Each group had six parallels. The 96-well plate was incubated in a static incubator at 37 °C for 24 h. The bacterial suspension was discarded, and the biofilm was washed with sterile PBS buffer three times; then, 150 µL 0.5% crystal violet staining suspension was added to each well and dyed for 15 min; crystal violet suspension was discarded, and each well was washed with PBS buffer. The biofilm was dried at room temperature, and finally, 33% acetic acid was used to dissolve crystal violet. The optical density at OD_570_ nm was measured to quantify biofilm formation (Ahmed et al. 2019).

#### Dispersion of pre-formed biofilm by L-HSL

One hundred fifty microliter bacterial suspension with a concentration of 1 × 10^7^ CFU/mL was added to a 96-well plate. The plate was incubated in a static incubator at 37 °C for 24 h. The supernatant was discarded, and the biofilm was gently washed three times with sterile PBS; then, 150 µL PPGAS medium containing 0, 10, 100, and 200 µM L-HSL, respectively, was added to each well in the 96-well plate. The plate was incubated in a static incubator at 37 °C for 24 h. After removing the culture medium, the plate was gently washed three times with sterile PBS. The remaining biofilm was quantified using the same method as described above. Each group made six parallels.

#### Effect of L-HSL on the structure of the biofilm

The sterile coverslips were placed in a 6-well plate, and a 2 mL 1 × 10^7^ CFU/mL bacteria suspension containing 0, 10, 100, and 200 µM L-HSL, respectively, was added. The plate was statically incubated at 37 °C for 3 days; then, the bacteria suspension was discarded, and the coverslips with biofilms were gently washed three times using sterile PBS. Next, the biofilms were fixed overnight using 2.5% glutaraldehyde suspension; the coverslips were further rinsed three times using sterile PBS and were dehydrated using a series of ethanol gradients, respectively (50%, 70%, 80%, 90%, 95%) for 15 min. Then, the coverslips were dehydrated twice using 100% ethanol for 20 min (Brandão et al. 2018). The coverslips were dried by a vacuum freeze dryer (INDEP Mini-E) and were observed by scanning electron microscope (SEM) at a magnification of × 4000 (GeminiSEM 500).

#### Extracellular polysaccharide assay

Bacterium was collected after cultivating for 24 h at 37 °C and 150 rpm in LB broth medium containing 0, 10, 100, and 200 µM L-HSL, respectively. Extracellular polysaccharide was extracted by heat extraction method (Chen et al. 2007). Bacterium were heated at 80 °C for 30 min and were centrifuged at 12,000 rpm and 4 °C for 15 min; then, the supernatant (extracellular polymers) was retained. The extracellular polysaccharide content was determined by the anthrone-sulfuric method (Ren et al. 2023), and extracellular polysaccharide yields were obtained from glucose standard curve calculations.

Take 5 mL of extracellular polymers extract, quickly freeze it at − 80 °C for 10 min, then lyophilize it for 24 h. Grind the sample with potassium bromide at a ratio of 1:100 (w/w), press it into a pellet, and use a Fourier transform infrared (FT-IR) spectrometer to analyze the surface chemical groups of the extracellular polymers (Nair et al. 2021).

### Motility assay

#### Swimming assay

The swimming assay method was modified from Yang et al. (2021); the swimming medium (LB agar medium containing 0.3% agar) was sterilized and cooled to approximately 50 °C; L-HSL (final concentrations: 0, 10, 100, 200 µM, respectively) was added. The mixture was used to prepare solid plates. Two microliter bacterial suspension (1 × 10^7^ CFU/mL) was inoculated at the center of each plate. After incubating at 37 °C for 24 h, the migration distance around the incubated spot was measured to assess the swimming assay.

#### Twitching assay

The twitching medium plates (LB medium containing 1.2% agar) containing 0, 10, 100, and 200 µM L-HSL, respectively, were prepared. The plates were inverted and incubated overnight. Then, sterile toothpicks were used to pick up the bacterial suspension (1 × 10^7^ CFU/mL). The toothpick was inserted into the center of the plate, penetrating through the agar to the bottom. After incubating at 37 °C for 48 h, the agar was discarded, and the plate was stained with 0.5% crystal violet for 10 min; then, excess crystal violet was washed off with sterile water, and the migration distance from the incubation spot was measured to assess twitching assay (Saqr et al. 2021).

### *C. elegans* survival assay

The infection model of *C. elegans* was established by referring to previous research methods with improvements (Cho et al. 2010). First, in a *C. elegans* growth medium (NGM) agar plate, the control group contained only 0.3% DMSO, while the experimental groups contained different concentrations of L-HSL (10, 100, and 200 µM). After inverting, the plates were left overnight. Then, 100 µL of *E. coli* OP50 bacterial suspension with a concentration of 1 × 10^9^ CFU/mL was spread onto the NGM plates. *E. coli* OP50 was used as the food source for *C. elegans*. The plates were incubated at 37 °C for at least 4 h to establish the bacterial lawn. Subsequently, 50 µL of *P. aeruginosa* bacterial suspension with a concentration of 2.8 × 10^9^ CFU/mL was evenly spread onto the prepared NGM plates. Here, NGM plates with both *E. coli* OP50 and *P. aeruginosa* served as the positive control, while NGM plates with only *E. coli* OP50 served as the negative control. All plates were incubated in a 37 °C incubator for 24 h to establish the bacterial lawn, then taken out and allowed to equilibrate to room temperature. Next, 100 synchronized L4 stage N_2_ *C. elegans* worms were transferred onto each plate. The plates were incubated at 20 °C, and observations were made every 8 h, recording the number of dead worms. The number of surviving worms and lost worms (due to crawling up the walls, burrowing, or other accidental deaths or disappearances) was also recorded. To prevent cross-contamination, dead worms were removed each time. Finally, the data of the surviving worms were tabulated to generate a survival curve.

### Transcriptomic analysis

#### Preparation of transcriptome sequencing samples

Bacterium were collected by centrifuge at 10,000 rpm and 4 °C for 15 min after cultivating for 24 h at 37 °C and 150 rpm in LB broth medium containing 0 and 200 µM L-HSL, respectively. Then, the cells were treated with 75% ethanol and incubated overnight at − 80 °C for inactivation. Afterward, the bacterial cell pellet was flash-frozen in liquid nitrogen for 10 min and immediately stored at − 80 °C.

#### Construction of a transcriptome sequencing database

The experiment utilized the TruSeq™ Stranded Total RNA Library Preparation Kit to construct the library. In the dNTPs reagent used for synthesizing the second strand of cDNA, dUTP was substituted for dTTP, allowing the second strand of cDNA to contain the bases A/U/C/G. Before PCR amplification, the second strand of cDNA was digested using the UNG enzyme to ensure that the library only contained the first strand of cDNA. The specific steps included (1) total RNA extraction: Total RNA was extracted from the sample, and its concentration and purity were assessed using Nanodrop2000. RNA integrity was checked using agarose gel electrophoresis, and RIN values were determined using Agilent2100. (2) Removal of rRNA. (3) mRNA fragmentation: Addition of fragmentation buffer facilitated random fragmentation of rRNA-depleted mRNA into fragments of approximately 200 bp. (4) cDNA synthesis: Using reverse transcriptase and random primers, a single-stranded cDNA was synthesized from mRNA templates. During the synthesis of the second strand, dUTP was substituted for dTTP in the dNTPs reagent, enabling the second strand of cDNA to contain A/U/C/G bases. (5) Adaptor ligation: The double-stranded cDNA had sticky ends, which were converted to blunt ends using End Repair Mix. Subsequently, an A base was added to the 3′ end for ligation with Y-shaped adaptors. (6) UNG enzyme digestion of cDNA second strand: Prior to PCR amplification, the UNG enzyme was used to digest the second strand of cDNA, ensuring that the library only contained the first strand of cDNA. (7) Sequencing.

#### Quality control and assembly of transcriptome data

Due to the presence of some low-quality data in the raw sequencing data from Illumina HiSeq, as well as a small fraction of reads containing artificial sequences such as sequencing primers and adapters, it is necessary to perform quality trimming on the raw data to improve the accuracy of subsequent assembly. This includes removing adapter sequences, trimming erroneous bases at the 5′ end (non-A, G, C, T), discarding reads with low sequencing quality at the 3′ end (< Q20), and removing reads with a proportion of N reaching 10%. Additionally, fragments with lengths less than 25 bp after adapter removal and quality trimming are eliminated, resulting in a set of high-quality reads, referred to as clean data, which are then used for genome assembly.

#### Gene expression analysis

Gene expression quantification was performed using RSEM (http://deweylab.github.io/RSEM/), and the quantification metric used was TPM (Transcripts Per Million reads). The DESeq2 package, Version 1.24.0, was employed to analyze the differential gene expression between samples, with a statistical significance set at a *P-*value < 0.05 and a fold change (FC) > 1.5 to identify significantly differentially expressed genes (DEGs). All identified genes were individually aligned to the following six major databases at the National Center for Biotechnology Information (NCBI): Nr, SWiss-Prot, Pfam, COG, GO, and KEGG, with an *E*-value < 0.05. To obtain gene ontology (GO) annotations and gene functional distributions for each UniGene cluster, the Blast2GO program and WEGO software were utilized (*E*-value < 0.05). Furthermore, the Kyoto Encyclopedia of Genes and Genomes (KEGG) automatic annotation server was used to search and assign each UniGene cluster to different pathways, providing insights into the functional implications of the identified genes.

#### Statistical analysis

All results are presented as mean ± standard deviation, and each experiment was repeated three times. GraphPad Prism 8 was used for graphing the results of the experiment, and the statistical analysis of nematode survival rate was performed using the log-rank test, while the rest of the data were analyzed using the *t*-test. *P* < 0.05 is considered statistically significant.

## Results

### Synthesis of compounds

After analyzing the product using ^1^H NMR, it was found to match the target product, confirming it as L-HSL. The ^1^H NMR results (Supplementary Fig. S1): (500 MHz, DMSO-d6) *δ* 7.68 (*d*, *J* = 8.0 Hz, 2 h), 7.49 (*d*, *J* = 8.0 Hz, 2 h), 4.57 (*dt*, *J* = 11.0, 8.5 Hz, 1 h), 4.34 (*td*, *J* = 8.9, 1.8 Hz, 1 h), 4.25–4.18 (*m*, 1 h), 3.60 (*s*, 2 h), 2.42–2.35 (*m*, 1 h), 2.17–2.10 (*m*, 1 h). The yield of the product was 80%.

### Growth curves

**Fig. 2:**
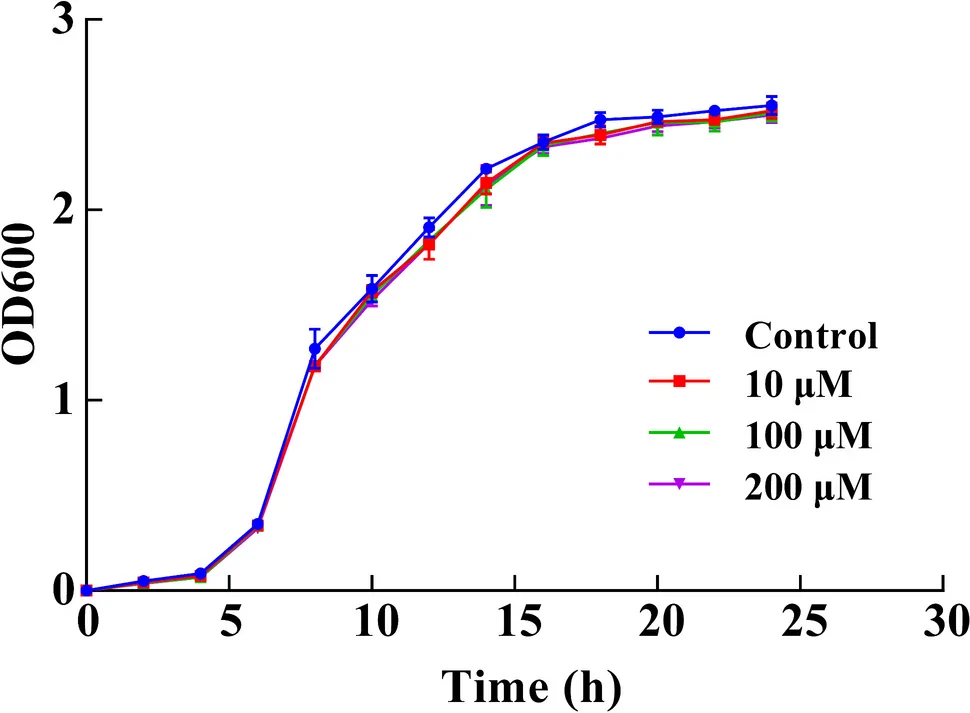
Effect of different concentrations of L-HSL on the growth curve of *P. aeruginosa*. Bacteria were cultured in a PPGAS medium at 37 °C and 150 rpm for 24 h under 0, 10, 100, and 200 µM L-HSL, respectively. OD_600_ nm was measured every 2 h

To assess the inhibitory effect of L-HSL on the biofilm formation and motility of *P. aeruginosa*, we first investigated the effect of L-HSL on the growth of *P. aeruginosa*. Figure 2 shows that L-HSL had no inhibition effect on the growth of *P. aeruginosa* at concentrations ranging from 10 to 200 µM.

### Effects of L-HSL on biofilm

**Fig. 3:**
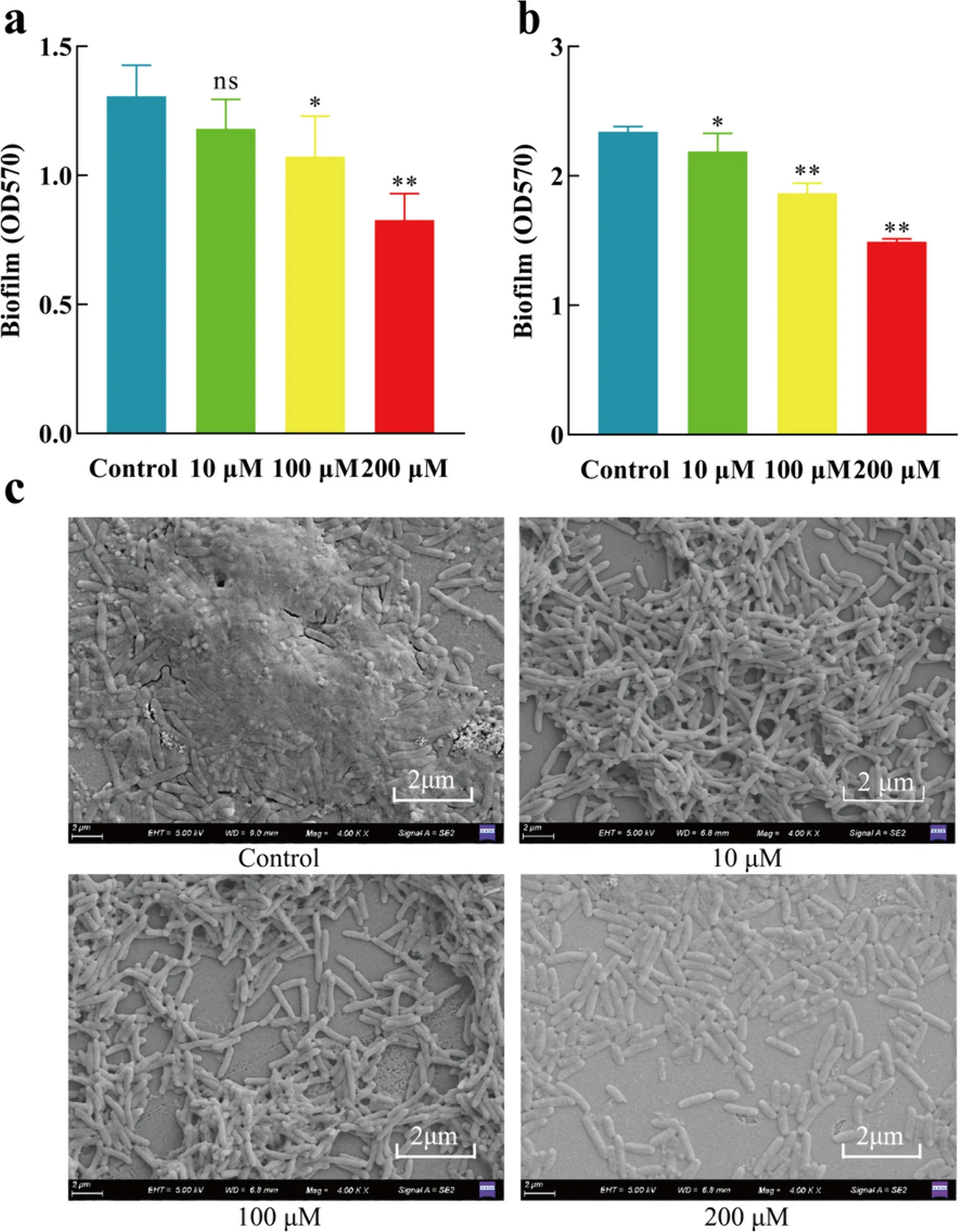
Effects of L-HSL on *P. aeruginosa* biofilm formation. **a** Effects of L-HSL on biofilm formation. **b** Effects of L-HSL on the dispersion of pre-formed biofilm. **c** Effects of L-HSL on the structure of biofilm. ns, no significant difference (*P* > 0.05). *Significant difference (0.01 < *P* < 0.05); **significant difference (*P* < 0.01)

Bacterial biofilm refers to a membrane-like structure formed by bacterial cells enveloped in their own secreted extracellular polymer matrix, adhering to solid surfaces or biological objects (Clauss et al. 2013). The formation of bacterial biofilm is a dynamic process that includes attachment, growth, maturation, and detachment stages (Rather et al. 2021). In this study, we investigated the effects of L-HSL on both the early formation process and the dispersion of pre-formed biofilm in *P. aeruginosa*. During the biofilm formation process (Fig. 3a), we found that inhibitory activities of L-HSL on biofilm formation were dose-dependent, and L-HSL had no significant impact on biofilm formation at the concentration of 10 µM (*P* > 0.05); however, in the presence of 100 µM L-HSL, a significant reduction in biofilm formation was observed (*P* < 0.05), and the biofilm formation was decreased nearly 36.71% at the concentration of 200 µM L-HSL (*P* < 0.05). Regarding the dispersion of pre-formed biofilm, Fig. 3b shows that the dispersion of pre-formed biofilm also was dose-dependent. At the concentration of 10 µM, L-HSL exhibited a slightly dispersing effect on the pre-formed biofilm (*P* < 0.05); however, the dispersion rate of pre-formed biofilm was nearly 36.27% at the concentration of 200 µM L-HSL. Finally, we visualized the effect of L-HSL on the structure of biofilm by SEM; the results are shown in Fig. 3c. The control group was covered with a thick polysaccharide matrix, with very few individual cells, indicating the presence of a substantial, well-developed biofilm. In contrast, at the concentration of 10 µM L-HSL, the glass slide surface showed less polysaccharide matrix and fewer biofilm structures, but the number of individual cells did not change significantly. At the concentration of 100 µM L-HSL, the biofilm was dispersed, the membrane-like structures were almost absent, and bacteria cells were released. At the concentration of 200 µM L-HSL, the biofilm was significantly dispersed, and the bacterial cells were fully released. In conclusion, these results indicated that L-HSL exhibited an inhibitory effect on biofilm formation and promoted the dispersion of the pre-formed biofilm of *P. aeruginosa*.

### Effect of L-HSL on extracellular polymers

**Fig. 4:**
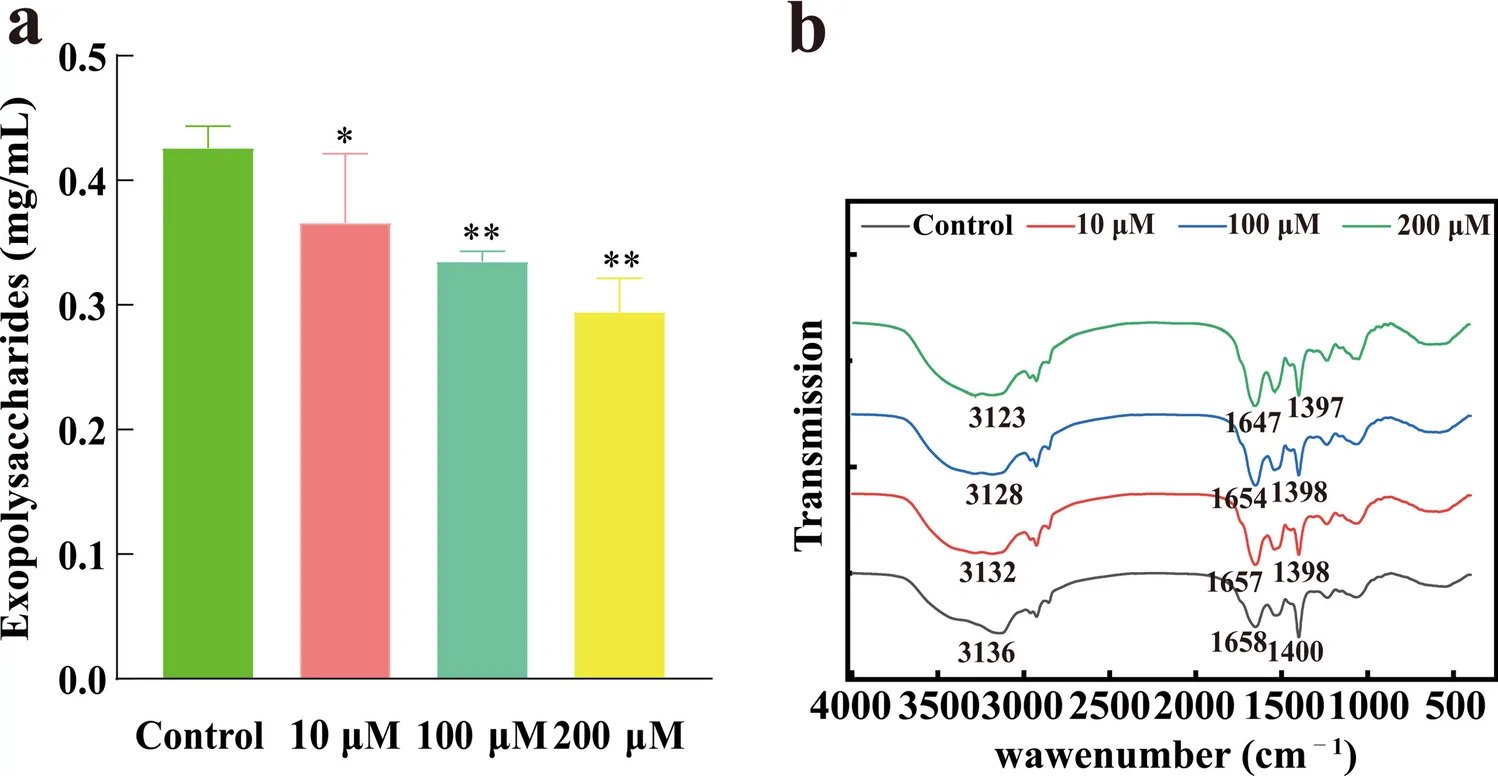
Effects of L-HSL on the surface chemical groups of extracellular polymers and extracellular polymers of *P. aeruginosa*. **a** Effects of L-HSL on extracellular polymers. **b** Effect of L-HSL on the surface chemical groups of extracellular polymers. ns, no significant difference (*P* > 0.05). *Significant difference (0.01 < *P* < 0.05); **significant difference (*P* < 0.01)

The production of extracellular polymers in *P. aerugino*sa was regulated by the QS system and played a promoting role in the initial formation and maturation of the biofilm, making it an essential component of the biofilm structure. QS inhibitors interfere with the production of pathogenic bacteria’s extracellular polymer. The results are shown in Fig. 4a. Compared with the control group, at the concentration of 100 µM L-HSL, extracellular polymer production was significantly inhibited (*P* < 0.01), and the inhibition rate of extracellular polymer production was nearly 30.84% at the concentration of 200 µM L-HSL.

FT-IR was used to analyze the surface groups of extracellular polymers, as shown in Fig. 4b. There are numerous absorption peaks in the extracellular polymers within the 500–4000 cm^−1^ range. Compared to the control group, the trend of the curves after treatment with 10 µM and 100 µM is almost identical, indicating a similar degree of influence on the extracellular polymer surface functional groups. However, 200 µM had a more significant impact. Specifically, the absorption peak at 3136 cm^−1^ is due to the O–H stretching vibration, indicating the presence of polysaccharides in the extracellular polymers. The peak at 1658 cm^−1^ is the amide I band C = O stretching vibration peak, which represents the secondary structure of proteins in membranes and flagella. The broadening of this peak suggests that the protein structure may have been damaged. The absorption peaks at 1330–1340 cm^−1^ are caused by the symmetric stretching vibration of the C = O bond, indicating the presence of carboxyl groups and some glycosides. The results suggest that L-HSL affects the electron-withdrawing functional groups such as O–H and C = O on the surface of extracellular polymers.

### Effects of L-HSL on the motility of *P. aeruginosa*

**Fig. 5:**
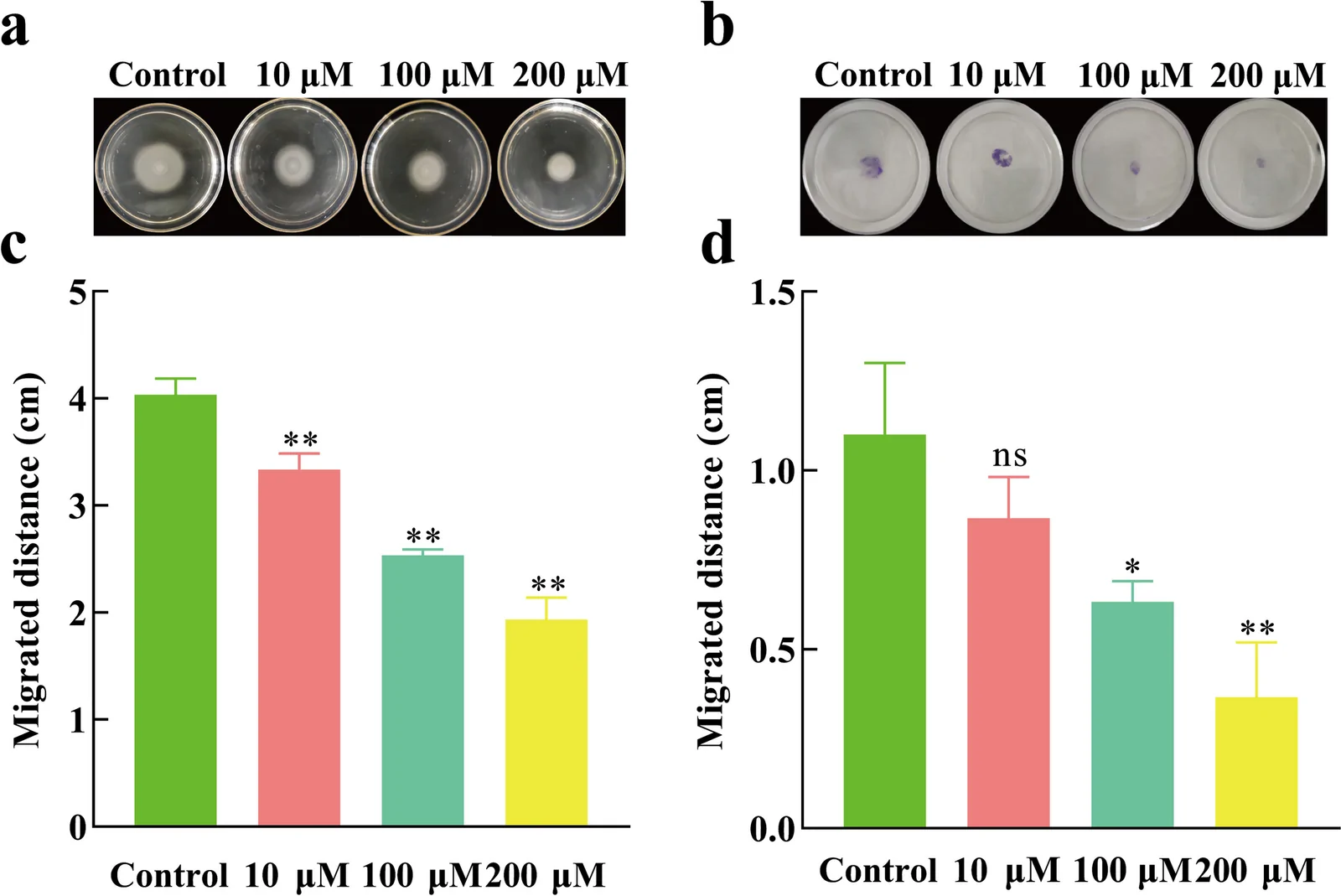
Effect of L-HSL on the motility of *P. aeruginosa*. **a** Swimming motility. **b** Twitching motility. **c** Swimming motility migrated distance. **d** Twitching motility migrated distance. ns, no significant difference (*P* > 0.05). *Significant difference (0.01 < *P* < 0.05); **significant difference (*P* < 0.01)

Motility plays a crucial role in the initial attachment process of bacteria; thus, controlling the potential of biofilm formation by inhibiting the motility of *P. aeruginosa* has become a powerful way to address bacterial resistance (Fleming et al. 2022). Bacterial swimming is motility mediated by a single terminal flagellum in a liquid environment or low-concentration agar medium (Al-Yousef et al. 2017)**.** As shown in Fig. 5c, swimming motility gradually decreases with increasing concentrations of L-HSL ranging from 10 to 200 µM. Figure 5a displays an image of swimming motility after 24 h of cultivation. Compared to the control group, at 48 h of cultivation, the swimming ability of *P. aeruginosa* was significantly weakened at 10 µM (*P* < 0.01), and at 200 µM, the diameter of the swimming is reduced to about 2 cm.

Bacterial twitching motility is mediated by type IV pili and is involved in the biofilm formation (An et al. 2006), as shown in Fig. 5d. Compared to the control group, twitching motility gradually decreases with increasing concentrations of L-HSL ranging from 10 to 200 µM. Unlike swimming motility, at 10 µM L-HSL, there was no significant effect on twitching (*P* > 0.05), but at 100 µM, there was a significant change (*P* < 0.05), and at 200 µM, twitching motility of *P. aeruginosa* was highly inhibited (*P* < 0.01). Figure 5b shows the images of twitching motility after 48 h of cultivation. Compared with the control group, *P. aeruginosa*’s twitching ability continuously weakened with varying concentrations of L-HSL. Overall, these results indicated that the motility of *P. aeruginosa* was inhibited by L-HSL.

### *C. elegans* survival assay

**Fig. 6:**
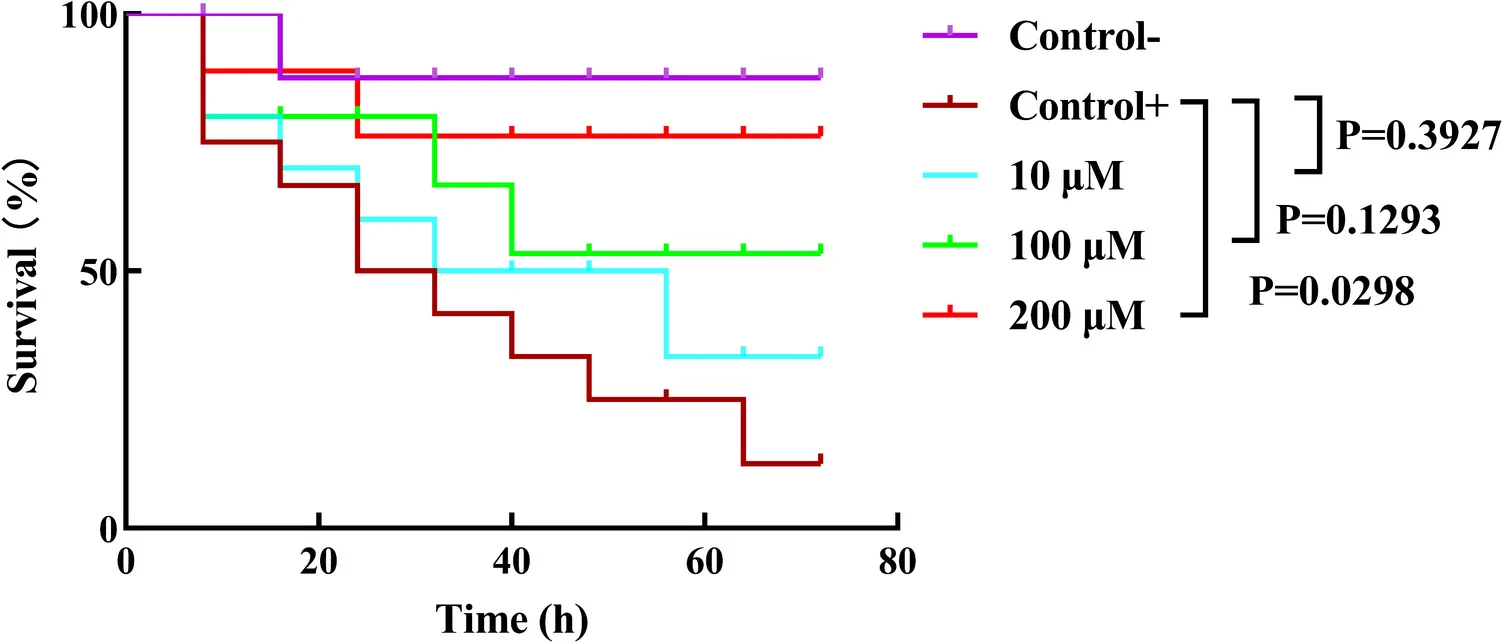
Effect of L-HSL on *C. elegans* survival assay. Nematode growth medium (NGM) plate, containing different concentrations of L-HSL (10, 100, and 200 µM), was inoculated with *E. coli* OP50 and *P. aeruginosa*. After culturing for 24 h, L4 stage *C. elegans* worms were transferred onto the plate. *C. elegans* survival assay was measured every 8 h

*C. elegans* is a small, transparent organism with a short life cycle, making it easy to culture and observe. It is an excellent model microorganism for assessing drug toxicity and determining survival rates after bacterial infection (Park et al. 2017). In the study, the *C. elegans* infection model was established to evaluate the protection effect of L-HSL on *P. aeruginosa* infection. The experimental results, as shown in Fig. 6, indicate that the survival rate of *C. elegans* significantly increased with the concentration of L-HSL ranging from 10 to 200 µM. Notably, at a concentration of 200 µM L-HSL, the worm survival rate increased by 71.78% compared to the positive control group after 72 h of incubation. These results suggest that L-HSL improves the survival rate of infected *C. elegans* in a dose-dependent manner.

### Transcriptome analysis

RNA quality, concentration, and integrity met the standard requirements for library preparation and transcriptome analysis (Table S1). The sequencing quality was good, and the obtained sequences were highly reliable (Table S2). Additionally, when the alignment rate of Uniq Mapped Reads exceeds 70%, it is generally considered suitable for subsequent analysis. Therefore, we aligned the Clean Reads, obtained after quality control, to the reference genome and found that the alignment results met the experimental requirements (Table S3). Finally, to avoid the chance occurrence of experimental results and enhance their credibility, we used the Pearson correlation coefficient to map the gene expression levels between different biological replicates. The results indicated a strong correlation and good reproducibility among the samples (Fig. S2).

**Fig. 7:**
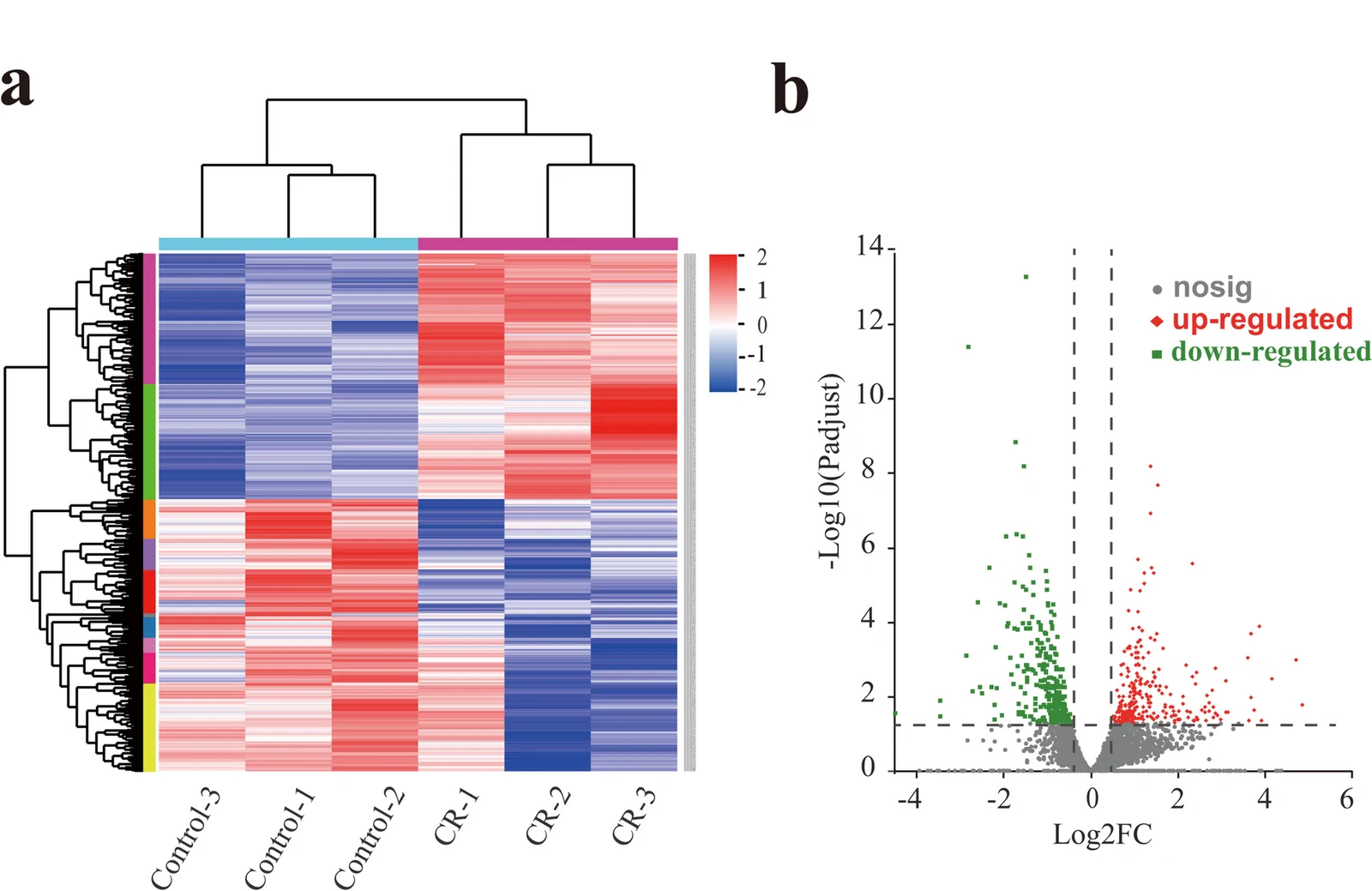
Differentially expressed gene map. **a** Differential expression genes clustering heat map. Red indicates upregulated genes, and blue indicates downregulated genes. **b** Differential expression gene volcano map

To study the changes in transcriptional levels of *P. aeruginosa* in response to L-HSL treatment, we analyzed the gene expression in the control group and the experimental group. The heatmap of DEGs is shown in Fig. 7a. Compared to the control group, L-HSL treatment led to both upregulation and downregulation of genes involved in the same biological processes, indicating that certain metabolic processes or cellular pathways may be involved in the formation of the biofilm. The volcano plot of DEGs (Fig. 7b) demonstrated that, compared to the control group, the log2FC values of gene expression levels after L-HSL treatment were mainly distributed within the range of − 4 to 4. There were a total of 539 significantly differentially expressed genes, including 242 upregulated genes and 297 downregulated genes (Table S4). This indicates that the presence of L-HSL has an impact on the formation of the *P. aeruginosa* biofilm.

The results are shown in Table 1: 5651 (99.89%) genes were aligned to the Nr database, 4264 (75.38%) genes were aligned to the SWiss-Prot database, 5154 (91.11%) genes were aligned to the Pfam database, 4880 (86.26%) genes were aligned to the COG database, 4636 (81.95%) genes were aligned to the GO database, and 3415 (60.37%) genes were aligned to the KEGG database.

**Table 1:** Results plot of the gene alignment analysis

	Total	Nr	SWiss-Prot	Pfam	COG	GO	KEGG
Number of unigenes	5657	5651	4264	5154	4880	4636	3415
Percent of unigenes (%)	100	99.89	75.38	91.11	86.26	81.95	60.37

**Fig. 8:**
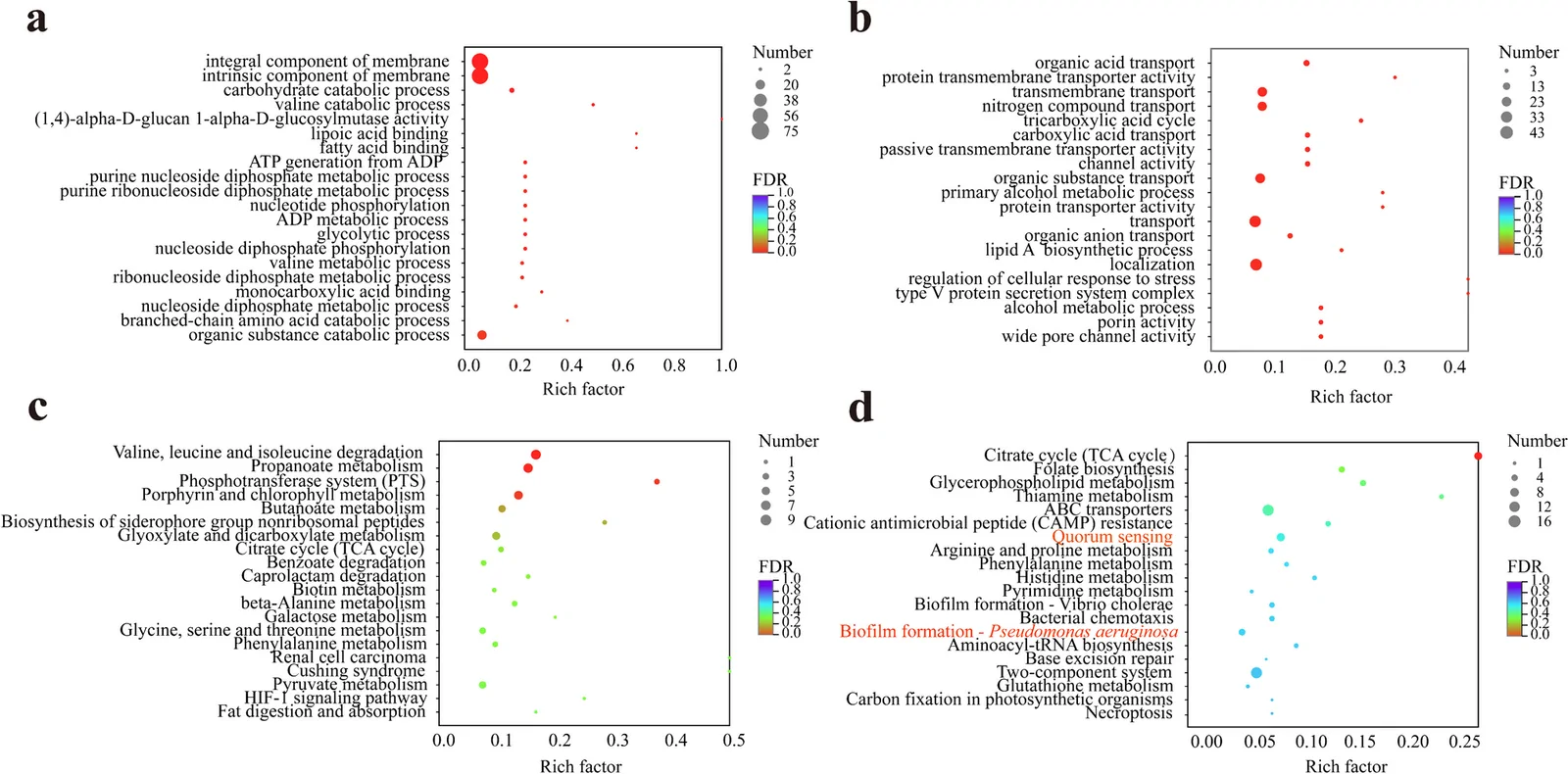
Enrichment analysis of the differentially expressed genes. **a** GO enrichment analysis of the upregulated genes. **b** GO enrichment analysis of the downregulated genes. **c** KEGG enrichment analysis of the upregulated genes. **d** KEGG enrichment analysis of the downregulated genes

GO (Gene Ontology) is a standardized gene function classification system used to describe the relationship between genes and gene products. It consists of three main categories: biological process, cellular component, and molecular function. In the study, a total of 4636 genes were annotated into the GO database, accounting for 81.95% of the total genes. Among them, 531 DEGs were mapped to the GO database: 207 genes were related to molecular function (103 upregulated, 104 downregulated), 269 genes were related to cellular component (138 upregulated, 131 downregulated), and 55 genes were related to biological process (24 upregulated, 31 downregulated). The top 20 cells with the highest enrichment level of upregulated GO (Fig. 8a) and the top 20 cells with the highest downregulation level (Fig. 8b) were listed, respectively. Upregulated mRNA was mainly enriched in the integral component of the membrane (75) and the intrinsic component of the membrane (75); secondly, there were organic substance catabolic processes (24), carbohydrate catabolic processes (7), and purine nucleoside diphosphate metabolic processes (4) in biological processes. The highest levels of downregulated mRNA enrichment were localization (43), transport (42), organic substance transport (30), transmembrane transport (29), and nitrogen compound transport (26). These GO enrichment analyses provided valuable insights into the functional characteristics of the differentially expressed genes and shed light on the molecular mechanisms influenced by L-HSL treatment in the context of cellular components, molecular functions, and biological processes.

The KEGG metabolic pathway database includes seven pathways: genetic information processing, cellular processes, environmental information processing, organismal systems, human diseases, metabolism, and drug development. In the experiment, a total of 3415 genes (60.37%) were annotated into the KEGG pathway. Compared with the control group, 1904 genes showed changes, resulting in 348 DEGs (174 upregulated,174 downregulated). Out of these 348 DEGs, 273 (108 upregulated, 165 downregulated) were annotated to six major pathways as follows (Table S5): metabolism: 157 (81 upregulated, 76 downregulated), genetic information processing: 16 (4 upregulated, 12 downregulated), environmental information processing: 51 (15 upregulated, 36 downregulated), cell processes: 31 (4 upregulated, 27 downregulated), biological systems: 7 (7 downregulated), and human diseases: 11 (4 upregulated, 7 downregulated).

Enrichment analysis of DEGs using KEGG was conducted. For upregulated mRNAs, a total of 63 enriched pathways were identified, and the top 20 pathways are shown in Fig. 8c. The five most enriched pathways for upregulated mRNAs were valine, leucine, and isoleucine degradation (9); propionate metabolism (8); porphyrin and chlorophyll metabolism (7); glyoxylic acid and dicarboxylate metabolism (6); and butanoate metabolism (5). For downregulated mRNAs, a total of 85 enriched pathways were identified, and the top 20 pathways are shown in Fig. 8d. The five most enriched pathways for downregulated mRNAs were ABC transporters (16); two-component systems (16); [Medicine] information exchange of QS bacteria (9); citrate cycle (8 cycles); biofilm formation—*P. aeruginosa* (6). Downregulated DEGs involved in the regulation of the QS system in *P. aeruginosa* are listed in Table 2: *GV84_RS03095*, *BGV84_RS03090*, *BGV84_RS19945*, *BGV84_RS10295*, *BGV84_RS08330*, *fadD1*, *BGV84_RS03100*, *crp*, and *potA* genes. Additionally, it was found that *BGV84_RS17725*, *tssC*, *tssC*, *tssB*, *BGV84_ RS09675*, and *crp* genes participate and are involved in the regulation of biofilm formation in *P. aeruginosa*. Notably, the *crp* gene is involved in the regulation of both quorum sensing and biofilm formation pathways*.*

**Table 2:** Differential expression gene of quorum sensing system

Gene ID	Gene name	Log2FC (CR/control)	Gene description
BGV84_RS03095	*BGV84_RS03095*	− 1.559781075	ABC transporter permease
BGV84_RS03090	*BGV84_RS03090*	− 1.680298592	ABC transporter substrate-binding protein
BGV84_RS19945	*BGV84_RS19945*	− 0.909253934	Branched-chain amino acid ABC transporter substrate-binding protein
BGV84_RS10295	*BGV84_RS10295*	− 0.698553072	3-Deoxy-7-phosphoheptulonate synthase
BGV84_RS08330	*BGV84_RS08330*	− 0.926776885	Phospholipase C%2C phosphocholine-specific
BGV84_RS08435	*fadD1*	− 0.606755484	Long-chain-fatty-acid–CoA ligase FadD1
BGV84_RS03100	*BGV84_RS03100*	− 1.268644375	ABC transporter permease
BGV84_RS03330	*crp*	− 0.799764769	cAMP-activated global transcriptional regulator CRP
BGV84_RS03085	*potA*	− 1.947836815	Polyamine ABC transporter ATP-binding protein
BGV84_RS17725	*BGV84_RS17725*	− 1.038754952	Hcp family type VI secretion system effector
BGV84_RS13315	*tssC*	− 0.777812462	Type VI secretion system contractile sheath large subunit
BGV84_RS00435	*tssC*	− 0.719802055	Type VI secretion system contractile sheath large subunit
BGV84_RS00430	*tssB*	− 0.85608708	Type VI secretion system contractile sheath small subunit
BGV84_RS09675	*BGV84_RS09675*	− 1.116569694	Bifunctional glycoside hydrolase 114/ polysaccharide deacetylase family protein

To further explore the mechanism of L-HSL’s action on *P. aeruginos*a, we performed GO and KEGG annotation and enrichment analyses of genes obtained from transcriptome sequencing. The KEGG enrichment analysis revealed that after L-HSL treatment, several genes involved in the QS pathway, including *GV84_RS03095*, *BGV84_RS03090*, *BGV84_RS19945*, *BGV84_RS10295*, *BGV84_RS08330*, *fadD1*, *BGV84_RS03100*, *crp* and *potA* genes, were significantly downregulated, Additionally, genes associated with the formation of the biofilm—*P. aeruginosa* pathway, including *BGV84_RS17725*, *tssC*, *tssC*, *tssB*, *BGV84_RS09675*, and *crp* genes, were significantly downregulated. ABC transporter protein permease is a highly hydrophobic membrane-integrated protein (Masulis et al. 2020) associated with drug resistance in biological biofilm. Enrichment analyses revealed that *BGV84_RS03095*, *BGV84_RS03090*, *BGV84_RS19945*, *BGV84_RS03100*, and *potA* are involved in different types of binding proteins and ABC transporters, suggesting that QS can affect multiple ABC transporters which actively transport certain substrates in and out of the cell to adapt to the biological biofilm.

## Discussion

*P. aeruginosa* is a common opportunistic pathogen in nature, and its infection is closely associated with the formation of host surface biofilm (Bucior et al. 2010). QS is involved in regulating bacterial biofilm formation by sensing population density. Bacterial biofilm is a protective mechanism formed by bacteria to adapt to adversity and is a complex multicellular three-dimensional structure. Studies have revealed that biofilm formation significantly increases the minimum inhibitory concentration (MIC) and minimum bactericidal concentration (MBC) of bacteria, often being 10–1000 times higher than those of planktonic cells (Høiby et al. 2011). Therefore, conventional antibiotics have become less effective in eradicating biofilms (Sharma et al. 2019), and the development of QS system inhibitors is a novel strategy to overcome bacterial biofilm formation (Hançer Aydemir et al. 2018).

Extracellular polysaccharides play a crucial role in bacterial colonization on abiotic or biological surfaces and the formation of bacterial biofilm. It mainly consists of extracellular polysaccharides, rhamnolipids, extracellular DNA (eDNA), and proteins (Costerton et al. 1999). Extracellular polysaccharides promote bacterial adhesion, which is a key step in the formation of biofilm (Ma et al. 2006). *P. aeruginosa* secretes three types of extracellular polysaccharides: Psl, Pel, and alginate (Ryder et al. 2007). During the initial stages of biofilm formation, Psl affects bacterial motility, while in the middle stages, it helps anchor bacteria within the biofilm structure. A reduction in Psl leads to a decrease in biofilm production (Ma et al. 2006). Pel, which is rich in glucose, provides the necessary sugar for biofilm formation (Friedman and Kolter 2004). Thus, inhibiting the production of bacterial extracellular polymers can inhibit biofilm formation, reduce bacterial resistance, and effectively prevent biofilm-associated bacterial infections. In this study, we investigated the impact of L-HSL concentrations ranging from 10 to 200 µM on *P. aeruginosa* biofilm formation. The results showed that L-HSL at 200 µM significantly inhibited biofilm formation and disrupted mature biofilm. Scanning electron microscopy visualized the mature biofilm structure, with the L-HSL-treated group showing biofilm dispersion and complete disappearance of the biofilm at 200 µM. To further confirm the effects of L-HSL on biofilm formation, we measured the extracellular polysaccharide content and changes in the surface chemical structure of extracellular polymers. The results demonstrated that at 200 µM L-HSL concentration, extracellular polysaccharide production was significantly reduced, and it affected the surface electron-accepting groups of extracellular polymers. These findings suggest that L-HSL can inhibit bacterial biofilm formation, and we propose that the reduction in extracellular polysaccharides is one of the reasons for the decrease in biofilm formation.

Bacterial surface motility also plays a role in influencing the formation and structure of biofilm (Zhang et al. 2014). *P. aeruginosa* possesses two types of motility appendages: flagella and type IV pili. Flagella mediate swimming movement, aiding bacterial cells in attaching to surfaces (Conrad et al. 2011). Once attached, cells form microcolonies through twitching motility, mediated by type IV pili, which promotes biofilm colonization and maturation (Marko et al. 2018). *P. aeruginosa* exhibits two modes of twitching motility known as “Walking” and “Crawling.” “Walking” demonstrates a form of superdiffusion with an unpredictable direction, and the transition from “Crawling” to “Walking” is facilitated by the contraction of type IV pili. Subsequently, the bacteria detach from the surface through flagellar rotation and swim and then reattach to new surfaces, forming new biofilms. Research has shown that bacterial motility is regulated by the QS system (Atkinson et al. 2006). Curcumin (Kumar et al. 2013) and N-acetylcysteine (Lima et al. 2023) are two different QS inhibitors that can inhibit the motility phenotype of *P. aeruginosa*. Notably, N-acetylcysteine demonstrated an 80% inhibition rate on swimming at a concentration of 10,000 µM. Inhibiting the QS system has been shown to reduce biofilm formation (Mayer et al. 2020). In this study, L-HSL significantly reduced both types of motility phenotypes in *P. aeruginosa* at a concentration of 200 µM. Therefore, we also consider that the reduction in motility phenotypes leads to a decrease in biofilm formation.

In addition, we investigated the therapeutic effects of L-HSL on the nematode model *C. elegans* by establishing an infection model. Our study revealed that L-HSL at concentrations between 10 and 200 µM increased the survival of infected nematodes.

The *crp* gene encodes the cAMP-activated global transcriptional regulator CRP, and cAMP acts as an intracellular second messenger. In *P. aeruginosa*, cAMP is synthesized by adenylate cyclases CyaA and CyaB, and it participates in the regulation of the QS system, T3SS, exotoxin A, protease IV, and type IV pilus biogenesis (Wolfgang et al. 2003; Coggan et al. 2022). Type IV pili are associated with *P. aeruginosa* twitching motility. Moreover, the *BGV84_RS10295* gene encodes 3-deoxy-7-phosphoheptulonate synthase, which is associated with extracellular polysaccharides. Microbial cells secrete extracellular polysaccharides after adhering to surfaces, promoting biofilm adhesion and the formation of microbial colonies and downregulating genes related to extracellular polysaccharides, which leads to reduced extracellular polysaccharide production, limiting biofilm formation, which is consistent with our in vitro results.

In conclusion, L-HSL can be considered a compound that antagonizes the QS system in *P. aeruginosa*. However, the specific mechanism by which it regulates the upregulation and downregulation of QS-related genes in *P. aeruginosa* requires further investigation. To determine whether L-HSL can be used for the clinical treatment of biofilm-associated bacterial infections, further studies involving animal infection models are needed to explore its therapeutic effects on infection models.

## Supplementary Information

Below is the link to the electronic supplementary material.

Supplementary file1 (PDF 227 KB)

## Data Availability

All the sequencing data involved in this paper have been uploaded to the SRA database under accession number PRJNA1080181 (http://www.ncbi.nlm.nih.gov/bioproject/1080181).
