# Supplementary material for: Effect of L-HSL on biofilm and motility of Pseudomonas aeruginosa and its mechanism
Source: Appl Microbiol Biotechnol. 2024 Jul 16;108(1):418. doi: 10.1007/s00253-024-13247-7 (PMC11252199; doi:10.1007/s00253-024-13247-7)
Supplement: Supplementary file 1 — Supplementary file1 (PDF 227 KB) [file 253_2024_13247_MOESM1_ESM.pdf]

Applied Microbiology and Biotechnology

Supplementary data

Effect of L-HSL on biofilm and motility of *Pseudomonas aeruginosa* and its mechanism

Deping Tang[1], Yanyan Lin[1], Huihui Yao[1], Yali Liu[1], Yanpeng Xi[1], Mengjiao Li[1], Aihong Mao[2]*

1. School of Biological & Pharmaceutical Engineering, Lanzhou Jiaotong University, Lanzhou，730070, Gansu, China;
2. Gansu Provincial Academic Institute for Medical Research, Lanzhou, 730050, Gansu, China)

Corresponding author. Aihong Mao

E-mail: maoaih@aliyun.com; Phone: +86(931)495-6896

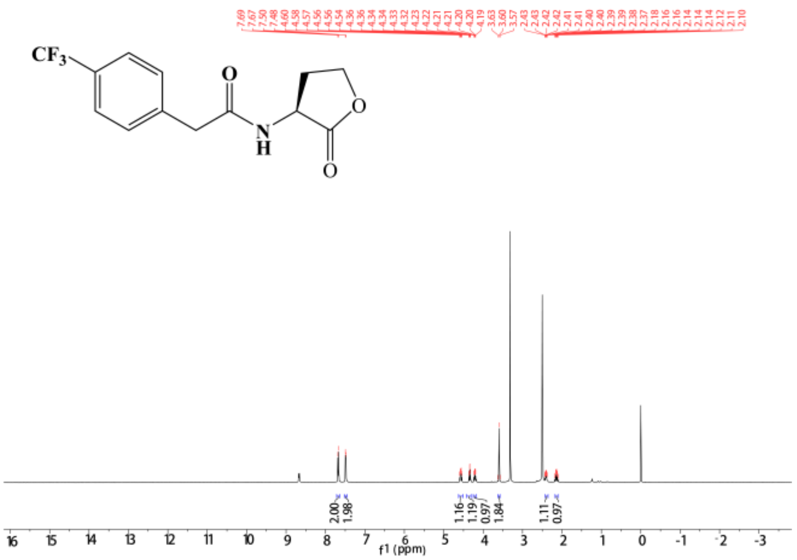

Fig. S1 L-HSL 1H-NMR (500 MHz, DMSO-d6).

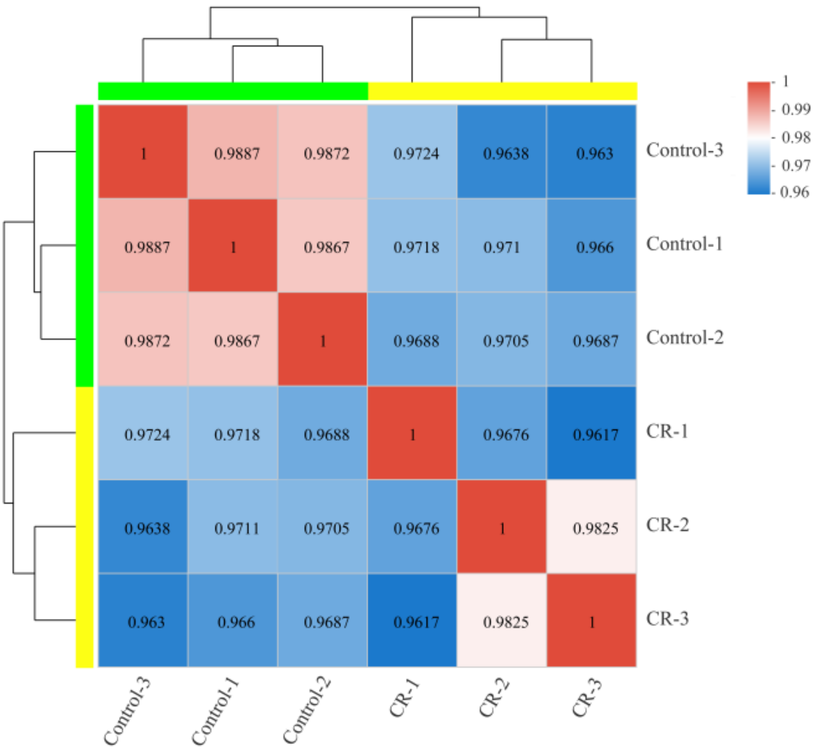

Fig. S2 Gene expression correlation plot of the samples.

Table S1 RNA quality evaluation results.

Sample name	Concentration (ng/μL)	total (μg)	OD 260/280	OD 260/230	RIN	Resuit
Control-1	210.60	7.37	2.09	2.21	9.80	A
Control-2	327.60	11.47	2.07	2.24	9.80	A
Control-3	340.90	11.93	2.06	2.22	9.80	A
CR-1	348.80	12.21	1.87	1.36	9.60	A
CR-2	171.50	6.00	2.07	1.56	9.60	A
CR-3	335.80	11.75	1.97	1.74	9.70	A

Table S2 Quality control results of sample sequencing data.

Sample	Control	CR
Raw reads	30355007	37944449
Raw Bases (bp)	4583606007	5729611849
Clean Reads	29845972	37438433
Clean Bases (bp)	3803811694	5049466684
Raw Q30 (%)	92.45	92.98
Clean Q30 (%)	94.75	94.49

Table S3 Alignment results of the samples to the reference genome.

Sample	Control	CR
Total Reads	29845972	37438433
Genome Mapped Reads	25415624(85.16%)	32522300(85.65%)
Unmapped Reads	4430348(14.84%)	4916133(14.35%)
Uniq Mapped Reads	24568630(82.32%)	23726650(69.28%)

Table S4 Significant differential gene expression statistics results.

Differentially expressed genes	Genes Number
Expressed mRNA and sRNA	539
Up-regulated mRNA and sRNA	242
Down-regulatedmRNA and sRNA	297
Expressed mRNA	508
Up-mRNA	241
Down-mRNA	267
Expressed sRNA	31
Up-regulated sRNA	1
Down-regulated sRNA	30

Table S5 DEGs anotation statistics of the KEGG.

First Category	Second Category	mRNA	up	down
Metabolism	Amino acid metabolism	33	17	16
	Biosynthesis of other secondary metabolites	1	1	-
	Carbohydrate metabolism	39	23	16
	Energy metabolism	15	5	10
	Lipid metabolism	15	6	9
	Metabolism of cofactors and vitamins	26	14	12
	Metabolism of other amino acids	7	3	4
	Metabolism of terpenoids and polyketides	7	6	1
	Nucleotide metabolism	6	1	5
	Xenobiotics biodegradation and metabolism	8	5	3
Genetic Information Processing	Folding, sorting and degradation	6	2	4
	Replication and repair	1	-	1
	Translation	9	2	7

Environmental Information Processing	Membrane transport	28	9	19
	Signal transduction	23	6	17
Cellular Processes	Cell growth and death	3	1	2
	Cell motility	5	-	5
	Cellular community - prokaryotes	21	3	18
	Transport and catabolism	2	-	2
Organismal Systems	Aging	1	-	1
	Endocrine system	4	-	4
	Environmental adaptation	1	-	1
	Nervous system	1	-	1
Human Diseases	Cancer: overview	1	1	-
	Cancer: specific types	1	1	-
	Drug resistance: antimicrobial	7	1	6
	Endocrine and metabolic disease	1	1	-
	Infectious disease: bacterial	1	-	1
